# Impact of COVID-19 in Female Urology

**DOI:** 10.1590/S1677-5538.IBJU.2020.S111

**Published:** 2020-07-27

**Authors:** Paulo Cesar Rodrigues Palma, Luiz Gustavo Oliveira Brito, Joanna Ghigo

**Affiliations:** 1 Universidade Estadual de Campinas Faculdade de Ciências Médicas Departamento de Cirurgia CampinasSP Brasil Divisão de Urologia, Departamento de Cirurgia, Faculdade de Ciências Médicas, Universidade Estadual de Campinas – UNICAMP, Campinas, SP, Brasil; 2 Universidade Estadual de Campinas Faculdade de Ciências Médicas Departamento de Obstetrícia e Ginecologia CampinasSP Brasil Divisão de Ginecologia, Departamento de Obstetrícia e Ginecologia, Faculdade de Ciências Médicas, Universidade Estadual de Campinas – UNICAMP, Campinas, SP, Brasil; 3 University of Malta Faculty of Medicine and Surgery Department of Obstetrics and Gynaecology Malta Department of Obstetrics and Gynaecology, Faculty of Medicine and Surgery, University of Malta, Malta

**Keywords:** Telemedicine, COVID-19 diagnostic testing [Supplementary Concept], Female

## Abstract

This review discusses the impact of COVID-19 in Female Urology, revises the most important disorders in this field and how their diagnosis and treatment may be modified due to the current pandemic. The text also discusses new options such as telemedicine and what clinical situations within Female Urology should be of utmost importance for the urologist to be careful about. We also discuss how surgeries are being postponed are resumed according to the local scenario.

## INTRODUCTION

Severe Acute Respiratory Syndrome Coronavirus (SARS-CoV2) is a pandemic disease that has contaminated nearly 4 million habitants worldwide and caused almost 300,000 deaths. It is a single-stranded RNA virus, with high infectivity rate, and although most of patients recover from disease, less than 20% of cases may worsen and need intensive care units for ventilatory support. Transmission occurs mainly by inhalation of droplets and aerosol containing the virus, but also through mouth, nose and eyes contact. Fecal--oral transmission may be possible. Laboratorial diagnosis can be done by RT-PCR (reverse transcriptase-polymerase chain reaction) and IgM/IgG antibody immunoassays, imaging is basically investigated by thoracic computerized tomography, and treatment is still controversial (1).

In summary, the pandemic caused by the Severe Acute Respiratory Syndrome Coronavirus (SARS-CoV-2) had a great impact not only for medical management, but also in doctor-patient relationship and for health care providers (2). Moreover, our patients present many known risk factors for COVID-19 (age over 60 years, hypertension, diabetes, etc) so their protection is essential.

## REPERCUSSION ON FEMALE UROLOGY

As female urology deals mostly with procedures related to quality of life, most of the these can be postponed. Telemedicine is an important tool, irrespective of whether the institution has a proper platform or if the doctor uses mobile applications and smartphones (3).

In Latin America, doctors will face many barriers such as: lack of massive testing for patients, , disparities between the number of COVID-19 patients admitted to public intensive care units (ICUs) versus private ICUs, under notification of data regarding infection and death rates and not enough personal protective equipment (PPE) (4).

Testing should be massively encouraged prior to surgery as well as revision of symptoms regarding COVID-19 with the patient. Within the operating room, N-95/FFP3 masks should be guaranteed for the surgical team (as well as the rest of accessories – shoe covers, gown, protective head covering, gloves and eye protection), a small number of people should be inside the operating room, and negotiation/discussion with the anesthesia team should be done prior to each procedure. For vaginal and abdominal surgeries, use of N-95 masks plus face shields should be present and smoke dispersion should be avoided as much as possible. In relation to laparoscopic procedures, discussion about pros and cons versus laparotomy should be done with the entire team, and extreme care should be taken to reduce pneumoperitoneum escape. Until time of writing, no data were available proving that COVID-19 viral particles were identified in surgical smoke (5).

## MANAGEMENT

In general, most urogynecological disorders can be treated conservatively (3). Data comparing telephone versus presential outpatient visit for postoperative floor disorders is already available (6). The most important disorders that lead patients to seek medical attention are mainly (7):

1. Urinary tract Infection (UTI)
2. Urinary incontinence (UI)
3. Pelvic Organ Prolapse (POP)

We shall also discuss about common procedures in urogynecology, such as urodynamics and cystoscopy. Table-1 comprises the complications potentially related to these disorders that may request an emergent visit to the hospital.

**Table 1:** Complications from main disorders in Female Urology that may demand a visit during COVID-19 pandemic.

Complications	Subtypes	Treatment*
Urinary tract infection	Relapsing UTI	Change antibiotics after requesting an urineculture
	Complicated UTI	Hospitalar management if patient present clinical instability with antibiotics and/or surgery
	Acute pelvic pain with urinary symptoms	Endovenous analgesics plus screening for differential diagnosis
Urinary incontinence	Postoperative acute retention	Clarify reasons and initiate treatment: sling mesh loosening or transurethral catheterization
	Postoperative urogenital fistula	Transurethral catheterization, prophylactic antibiotics and schedule surgery after pandemic
Pelvic organ prolapse	Bleeding or pain secondary to vaginal pessary	Pessary removal with or without sedation
	Vaginal vault rupture after POP surgery	Surgical vaginal vault closure
	Bladder eversion	Manual reduction with or without surgical management

* If surgical treatment, preoperative recommendation would be to test all patients if COVID-19 kits available, or to prioritize oligosymptomatic or symptomatic patients. Postoperative recommendation would be for same-day discharge and to consider prophylaxis or treatment of anticoagulation for symptomatic COVID-19 cases.

### Urinary tract infection (UTI)

Women with UTI symptoms should initially be managed by remote communication. The most prevalent and predictive symptoms are dysuria, worsening frequency or urgency, gross hematuria (8). Presence of vaginal symptoms reduces the predictive value for UTI. In a grossly scenario, clinical history allows the female urologist to separate the complicated presentation from non-complicated UTI.

Acute bacterial cystitis should be managed empirically as we will not have access, in many facilities, to guided-antibiograms. Empiric treatments with either trimethoprim-sulfamethoxazole (TMP-SMZ) or nitrofurantoin are cost-effective choices. Some options for uncomplicated UTI are TMP-SMZ 160/800 mg orally twice for three days (if local antibiotic resistance rate is not exceeding 20%), nitrofurantoin 100 mg orally twice daily for 5 days or fosfomycin trometamol 3 g once. If the patient does not improve or diagnosis in unclear to be considered by telemedicine, a urine sample may be left at the clinic for urinalysis, and if positive, a sample may be sent for culture and sensitivity (3).

### Urinary incontinence (UI)

For initial appointments, telemedicine allows history-taking. One day prior to consultation, the short-form questionnaires may be sent by email to improve consultation, or patient may answer the questionnaire guided by a nurse or chaperone prior to consultation at the same day. We must consider the normal scenario of an outpatient clinic; thus, in general, UI will be probably divided into stress, urgency and mixed. Other forms will probably require a presential visit. In mixed urinary incontinence, direct treatment towards the predominant symptom.

Conservative therapy is the first line therapy, and the alignment with a physical therapy improves resolution of each case. Pelvic floor muscle exercises, Kegel exercises, timing voiding should be mainly conducted on-line. At present there are many apps like iPelvis and others that allow for home practicing.

Secondary referral must be considered for some specific symptoms such as: gross hematuria, persistent bladder or urethral pain, previous continence surgery with pain and/or recurrent UT and voiding difficulties, including urinary retention.

Two COVID-19 symptoms may merge or cause confusion in differential diagnosis – dry cough with no underlying cause (pulmonary baseline disorder) and diarrhea – if patients start to present these symptoms, associated with fever, local testing should be provided.

Patients with severe urgency may be managed with antimuscarinics or beta 3 adrenergics. It is important to explain the side effects, success rate and the need for adhesion, which is typically low. Oxybutinin should be avoided in the elderly, antimuscarinics should be excluded for disorders such as closed-angle glaucoma. Patients should be aware that any major side effect should make them contact the hospital for referral and/or further instructions. One side effect (SE) from solifenacin is QT prolongation during electrocardiogram. Thus, for a patient with COVID-19 symptoms treated in an institution that might consider using drugs such as chloroquine, this must be discussed with the physician prior to initiating treatment. If patient needs to be referred to a hospital, she should carry a copy of her prescription.

### Pelvic organ prolapse (POP)

Patients with POP should initially be managed by remote communication. Specific questionnaires are available, and it is important to calm the patient regarding the evolution of POP. Most of the cases do not present POP worsening in 9-12 months of follow-up (9).

**Figure 1:**
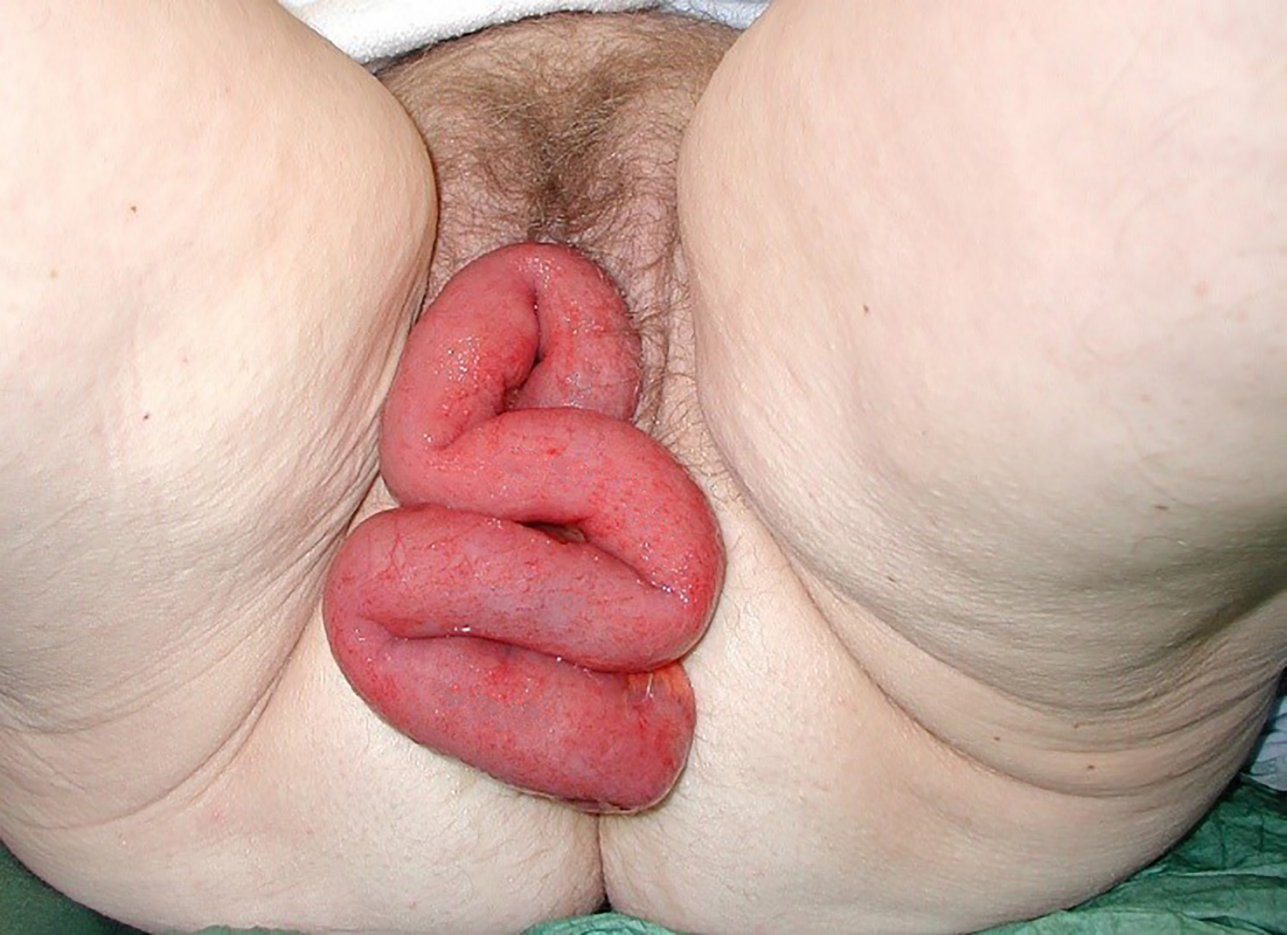
Rupture of vaginal vault prolapse.

Facilities for virtual communication can vary and include telephone/video conferencing. If prolapse is mild, patient should be advised to perform pelvic floor muscle training since there is no strong evidence that it can reduce POP, it may increase pelvic consciousness (10). On the other hand, if there is a large bulge affecting bladder and bowel emptying and/or in presence of ulceration, a face-to-face appointment will be required. Follow-up of surgical procedures can be carried out virtually using telephone or video conferencing with questionnaires focusing on patient reported outcomes (satisfaction, subjective improvement) but if a reason to see patient is identified, a face-to-face appointment may be the only option. If so, recommended PPE should be worn. In rare circumstances, POP may complicate as rupture of vaginal vault prolapse (Figure-1) or bladder eversion and surgery is recommended immediately.

**Figure 2:**
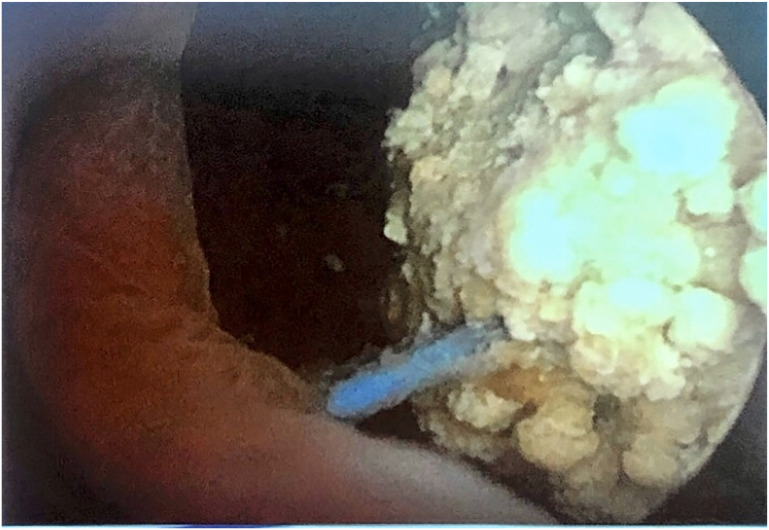
Cystoscopy in a patient with dysuria and interrupted flow. Notice the blue thread (needle suspension) and the stone.

Urodynamics and cystoscopy (Figure-2) are common procedures used in female urology. Cystoscopy is indicated when patients have symptoms or UTI after mid-urethral sling, to rule out bladder or urethral perforation and secondary stone formation. Because there is a risk of COVID negative get nosocomial infection, the use of trans labial non-invasive ultrasound might be considered.

Regarding urodynamics, as COVID-19 may have fecal transmission and a rectal balloon is used to indirectly measure abdominal pressure for calculating detrusor pressure, uro-dynamic should be postponed for six months or until it is safe to be done. Most of the recent guidelines for resuming elective procedures or surgery are suggesting observing local scenario (low numbers of infected people or consecutive reduction of the number of cases for at least, 14 days) (11).

## References

[1] 1. Liu J, Liu S. The management of coronavirus disease 2019 (COVID-19). J Med Virol. 2020. [published online ahead of print, 2020 May 5]. 10.1002/jmv.25965 PMC7267323 32369222

[2] 2. Hamza M, Khan HS, Sattar ZA, Hanif M. Doctor-patient communication in surgical practice during the coronavirus (COVID-19) pandemic. Br J Surg. 2020; 107:e193. 10.1002/bjs.11661 PMC7267138 32364265

[3] 3. Grimes CL, Balk EM, Crisp CC, Antosh DD, Murphy M, Halder GE, et al. A guide for urogynecologic patient care utilizing telemedicine during the COVID-19 pandemic: review of existing evidence. Int Urogynecol J. 2020:1–27. 10.1007/s00192-020-04314-4 PMC7185267 32342112

[4] 4. Brito LGO, Ribeiro PA, Silva-Filho AL; Brazilian Federation of Gynecology and Obstetrics Associations Gynecological Surgery Group for COVID-19. How Brazil Is Dealing with COVID-19 Pandemic Arrival Regarding Elective Gynecological Surgeries. J Minim Invasive Gynecol. 2020: S1553-4650; 30216-8. 10.1016/j.jmig.2020.04.028 PMC7194702 32344034

[5] 5. Mowbray NG, Ansell J, Horwood J, Cornish J, Rizkallah P, Parker A, et al. Safe management of surgical smoke in the age of COVID-19. Br J Surg. 2020. [published online ahead of print, 2020 May 3] 10.1002/bjs.11679 PMC7267397 32363596

[6] 6. Thompson JC, Cichowski SB, Rogers RG, Qeadan F, Zambrano J, Wenzl C, et al. Outpatient visits versus telephone interviews for postoperative care: a randomized controlled trial. Int Urogynecol J. 2019; 30:1639-46. 10.1007/s00192-019-03895-z PMC6699921 30783704

[7] 7. Thakar R. Guidance for the Management of Urogynecological Conditions During the Coronavirus (COVID-19) Pandemic. IUGA. [Internet]. Available at. https://www.iuga.org/news/message-from-the-president-guidance-for-themanagement-during-covid-19>. (accessed April 08, 2020).

[8] 8. Mishra B, Srivastava S, Singh K, Pandey A, Agarwal J. Symptom-based diagnosis of urinary tract infection in women: are we over-prescribing antibiotics? Int J Clin Pract. 2012; 66:493-8. 10.1111/j.1742-1241.2012.02906.x 22512608

[9] 9. Pizarro-Berdichevsky J, Borazjani A, Pattillo A, Arellano M, Li J, Goldman HB. Natural history of pelvic organ prolapse in symptomatic patients actively seeking treatment. Int Urogynecol J. 2018; 29:873-80. 10.1007/s00192-017-3450-0 28840270

[10] 10. Duarte TB, Bø K, Brito LGO, Bueno SM, Barcelos TM, Bonacin MA, et al. Perioperative pelvic floor muscle training did not improve outcomes in women undergoing pelvic organ prolapse surgery: a randomised trial. J Physiother. 2020; 66:27-32. 10.1016/j.jphys.2019.11.013 31843420

[11] 11. [No authors]. Local Resumption of Elective Surgery Guidance. American College of Surgeons. [Internet]. Available at. <https://www.facs.org/covid-19/clinicalguidance/resuming-elective-surgery>. (accessed April 17, 2020).

